# Mechanical strength and hydrostatic testing of VIVO adhesive in sutureless microsurgical anastomoses: an ex vivo study

**DOI:** 10.1038/s41598-021-92998-z

**Published:** 2021-06-30

**Authors:** Marius Heitzer, Julia Brockhaus, Kristian Kniha, Felix Merkord, Florian Peters, Frank Hölzle, Evgeny Goloborodko, Ali Modabber

**Affiliations:** 1 Department of Oral and Maxillofacial Surgery, University Hospital of RWTH Aachen, Pauwelsstraße 13, 52074 Aachen, Germany; 2 Institute of Textile Technology, RWTH Aachen University, Aachen, Germany

**Keywords:** Translational research, Biotechnology, Medical research

## Abstract

Conventional anastomoses with interrupted sutures are challenging and inevitably associated with trauma to the vessel walls. The goal of this study was to evaluate a novel alternative adhesive-based suture-free anastomosis technique that uses an intraluminal stent. Overall, 120 porcine coronary vessels were analyzed in an ex vivo model and were examined for their mechanical (n = 20 per cohort) and hydrostatic strength (n = 20 per cohort). Anastomoses were made using the novel VIVO adhesive with an additional intraluminal nitinol stent and was compared to interrupted suture anastomosis and to native vessels. Sutureless anastomoses withstood pressures 299 ± 4.47 [mmHg] comparable to native vessels. They were performed significantly faster 553.8 ± 82.44 [sec] (p ≤ 0.001) and withstood significantly higher pressures (p ≤ 0.001) than sutured anastomoses. We demonstrate that the adhesive-based anastomosis can also resist unphysiologically high longitudinal tensile forces with a mean of 1.33 [N]. Within the limitations of an in vitro study adhesive-based suture-free anastomosis technique has the biomechanical potential to offer a seamless alternative to sutured anastomosis because of its stability, and faster handling. In vivo animal studies are needed to validate outcomes and confirm safety.

## Introduction

For decades, sutured anastomoses have been the gold standard in microsurgical interventions, although they require lengthy operating times and high surgical skills. Yet, they are still prone to errors^1^. The use of sutures in small fragile vessels is inevitably associated with trauma to the vessel^2^. Therefore, many novel techniques and microvascular devices, such as absorbable couplers^3^, and magnetic compression rings^4^ have been investigated. Nevertheless, the use of such anastomoses is limited to anastomoses in veins^3,4^.

Different adhesive-based anastomosis procedures have recently been developed in microsurgery, including anastomosis using cyanoacrylate adhesives^5^ or fibrin-glues^6^. These procedures led to easier and faster anastomosis. So far, these adhesives have been used for anastomoses in preclinical studies but have not found clinical applications. Furthermore, fibrin adhesives are increasingly used clinically as a sealant for sutured anastomoses. Compared to other non-sutured microvascular anastomosis techniques, such as coupling devices, the use of fibrin glue in combination with sutures for anastomosis with a leakage rate of 6.3% is inferior to these methods^7^.

Intermolecular forces such as hydrogen bonds, electrostatic interactions, and van der Waals interactions are beneficial for adherence in dry tissues^8,9^. On the other hand, water prevents the interaction and separates the molecules of the two surfaces^10,11^. Tissue adhesives showed potential advantages over anastomoses by suturing or stapling^12,13^ but still suffer from limitations such as weak bonding, poor mechanical interactions with wet tissue, and low biocompatibility^12–16^. Therefore, the application of adhesives on wet tissues is considered particularly challenging^17^. In contrast, VIVO has shown significant hemostatic results in mass bleeding porcine liver resection models^18^ and long-term rabbit mass bleeding liver resection models^19^. Both investigations were conducted in liver tissue that was bleeding heavily and moist. These studies showed a good connection between the adhesive and tissue at the histological level that has not yet been metrically validated.

The use of in vitro models to train anastomoses^20^ or study new anastomotic procedures and to quantify biomechanical properties of anastomoses is common before these procedures are tested in an animal model^1,2^. Experimental analysis of the mechanical force by traction tests^21^ as well as the burst pressure tests^2^ are established in vitro methods for the evaluation of new anastomosis procedures.

In this study, we propose an alternative anastomosis technique. It uses the biodegradable tissue adhesive VIVO and an individualized expandable stent, similar to stents in coronary angioplasty, for intraluminal stabilization. We compared this technique to anastomoses with interrupted sutures and native vessels. Tests were conducted in a porcine ex vivo model on isolated coronaries. The goal of this ex vivo study was to evaluate the material and mechanical properties of seamless adhesive-based microvascular anastomoses in a new pressure testing and tension measurement. Those properties were compared to the established suture-based anastomoses in porcine ex vivo arterial vessels under standardized conditions.

## Methods

A total of 120 vessels were tested in this study. All surgical procedures were executed by a single and experienced person. Anastomoses were performed using the novel VIVO adhesive with an additional intraluminal nitinol stent (n = 40). That procedure was then compared to the gold standard, interrupted suture anastomosis (n = 40), and native vessels (n = 40). The burst pressure test was analyzed on a total of n = 60 vessels, corresponding to n = 20 native vessels in the control group, n = 20 vessels in the VIVO, and n = 20 vessels in the sutured anastomosis group. To determine the tensile force, experiments were conducted on n = 60 vessels, corresponding to n = 20 vessels per group. The vessel characteristics for all groups and tests are summarized in Table 1.

**Table 1:** General Data of vessels. NA, not applicable.

Groups	Vessels (n = 120)	Tensile strength determination External diameter (mm) Mean ± SD (n = 20)	Burst pressure tests External diameter (mm) Mean ± SD (n = 20)	Suture	Stiches
C (n = 40)	Porcine coronary vessels	3.09 ± 0.22	3.11 ± 0.21	NA	NA
Suture (n = 40)	Porcine coronary vessels	3.09 ± 0.16	3.09 ± 0.26	8/0	12
VIVO (n = 40)	Porcine coronary vessels	3.05 ± 0.25	3.04 ± 0.16	NA	NA

### Coronary arteries

Porcine coronary arteries represent an established model for microvascular anastomoses^22^. After approval by the official veterinarian, fresh pig hearts were obtained from a local slaughterhouse within 10 min after death and transported in a modified Krebs–Ringer bicarbonate solution at 4°C^23^. The vessels were harvested on cooled dissection tables under a surgical microscope (Carl Zeiss Meditec AG, Jena, Germany) and continually kept in a cold modified Krebs buffer solution^24^. The buffer was replaced every 10 min. Side branches of the coronary vessels were clipped with microvascular clips (Weck, New York, USA); small branches were ligated using 6/0 Vicryl threads (Ethicon, Hamburg, Germany). Vessels with a length of 22 mm were dissected to ensure the vessels’ comparability and a firm fit of the stents (Table 1). The coronaries were randomized into three groups: Unchanged original vessel stumps served as controls (C). Vessels in the suture-based group (Suture) were anastomosed after specimens were halved in the middle perpendicular to the central axis and readapted with 12 single sutures of Ethilon 8/0 (Ethicon, Hamburg, Germany). VIVO anastomoses were generated using VIVO and an auto-expanding stent as an intraluminal connection device. The stent was inserted using continuous circular pressure with microsurgical tweezers. After releasing the stent from the tweezer tips, the stent’s auto-expanding properties stabilized the vessels through its radial forces; vessel ends were adapted carefully without gaps. VIVO was applied on the outside of the vessel stumps. All vessels were tested immediately after preparation, and the time required for the completion of the anastomosis was recorded.

### Adhesive

The polyurethane-based, biodegradable adhesive, VIVO (Adhesys Medical GmbH, Aachen, Germany), was used in combination with a stent for anastomosis. VIVO consists of two synthetically-produced components: (1) polyurethane prepolymer with reactive isocyanate groups and (2) an amino-based curing agent.

### Stent

**Figure 1:**
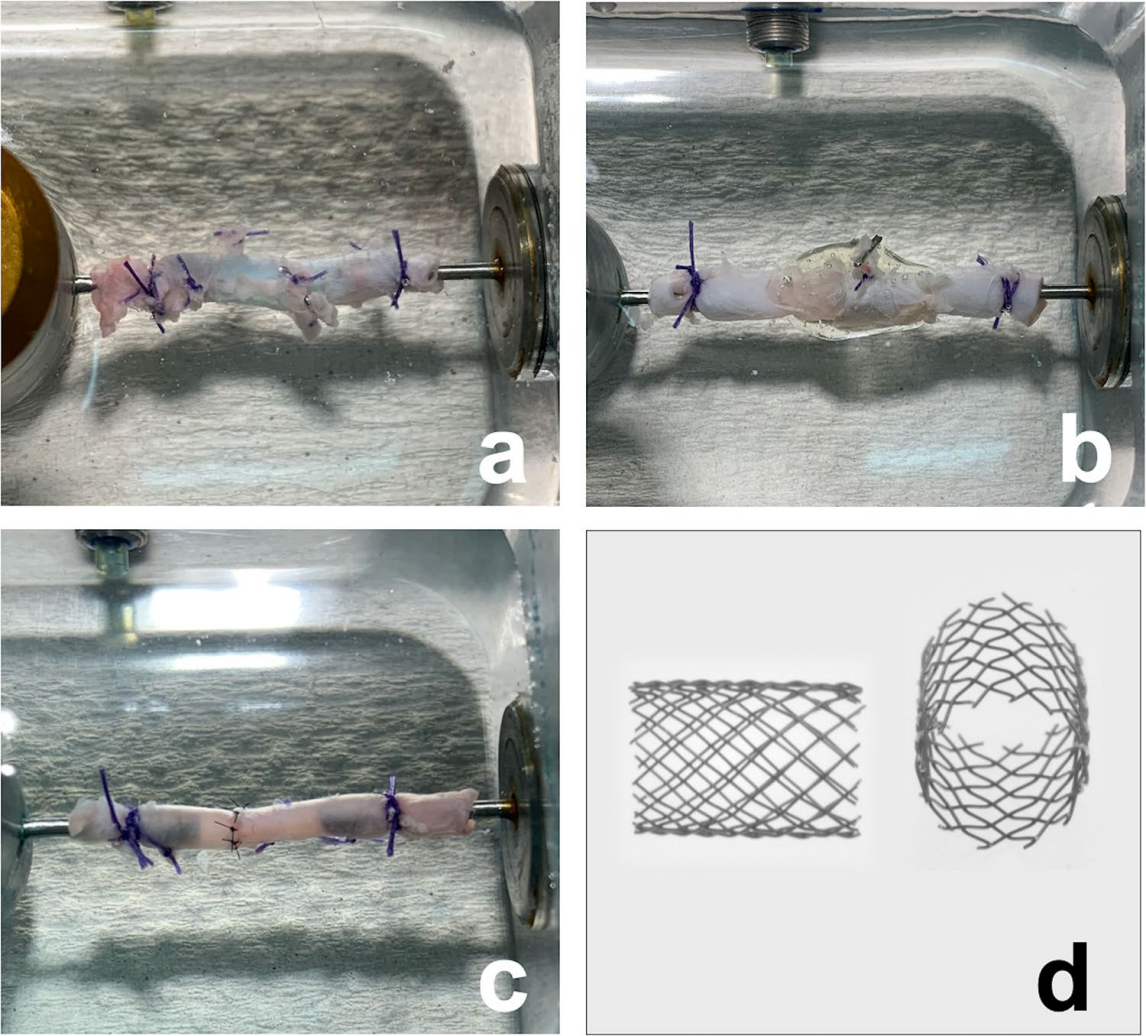
Vessels fixed in pressure apparatus. The native vessels served as control. Side branches were ligated (**a**). The Anastomosis made of VIVO and stent and the transparent nature of the adhesive allows control of the anastomosis region (**b**). Sutured anastomoses are the gold standard in microsurgery (**c**). Enlarged images of the stent design are shown in two perspectives (**d**).

The Stents (Fig. 1) were made of 100 µm diameter Nitinol#1 wire (Fort Wayne Metals, Castlebar, Co. Mayo, Ireland) and braided using a 24-wire braiding process on a maypole braiding machine (Circular Braiding Machine HS80/48, Körting successor Wilhelm Steeger GmbH & Co. KG, Germany). A mandrel (diameter 3 mm, length per stent 8 mm) was used in the multi-thread braiding process. A braiding angle of 45° was obtained using a 1:1–1 braiding pattern. The Nitinol framework was heat-set at 450 °C for 10 min using ambient atmosphere. The development and production of stents was done by Institut für Textiltechnik of RWTH Aachen University, Germany.

### Burst pressure tests

**Figure 2:**
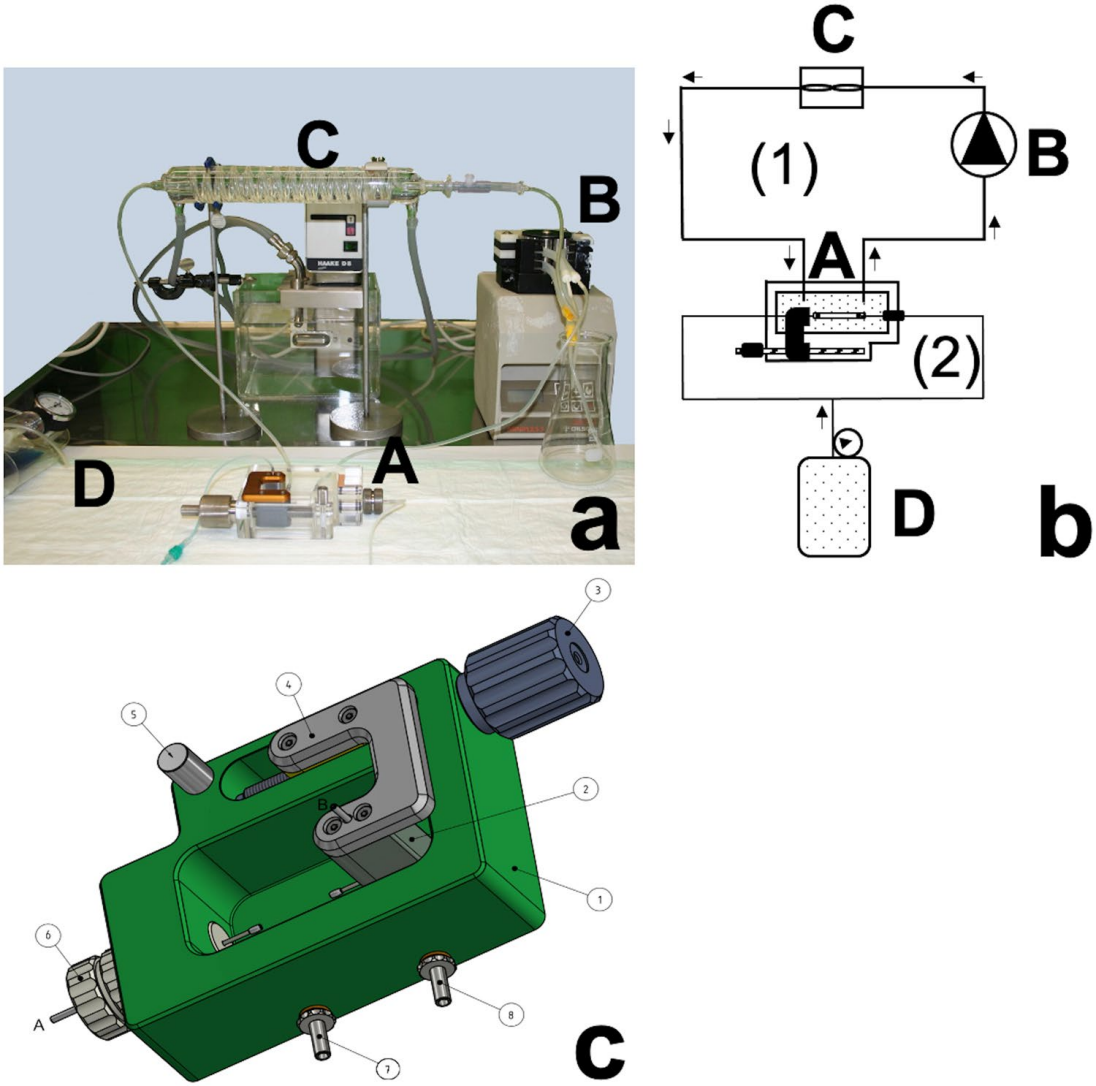
The structure of the equipment for the burst pressure tests. A Photograph of the technical setting (**a**) and schematic (**b**) of the pressure apparatus illustrate the experimental setup: (**A**) Pressure apparatus, (**B**) Peristaltic pump, (**C**) External heating coil, (**D**) Infusion pressure cuff. The schematic was drawn in Microsoft PowerPoint version 16.49 (Microsoft Corporation, Redmond, USA) running on Apple OS X. Schematic of the pressure apparatus (**c**): 1 Test tank; 2 Pressure inlet B / Button cannula holder length variable adjustable; 3 Set screw; 4 Bridge aluminum; 5 Sliding bolt; 6 Pressure inlet A / Button cannula holder; 7 Inflow; 8 Outflow. The pressure apparatus was designed in Inventor Professional 2014 (Autodesk, San Rafael, USA Windows) running on Windows 8.

The test system (Fig. 2) was developed following established publications^23,24^. It consisted of two circuits: The first circuit contained a custom-made pressure apparatus continuously circulated with a Krebs buffer at 37 °C and 7.4 pH^24^. The pressure apparatus (A) was designed in Inventor Professional 2014 (Autodesk, San Rafael, USA Windows) was produced in the institute workshop of the RWTH Aachen University (Aachen, Germany). The transparent buffer was tempered by an external interposed thermo-bath (C) (Haake D8, Thermo Haake GmbH, Karlsruhe, Germany). The exchange of liquid was ensured using an intermediate peristaltic pump MINIPULS 3 (B) (Abimed Gilson Incorporated, Middleton, USA) with an additional Krebs buffer reservoir. Once the vessel was fixed with two 6/0 Vicryl threads (Ethicon, Hamburg, Germany) at incorporated button cannulas (Hartmann Peha, Paul Hartmann AG, Heidenheim, Germany), the circuit (2) was closed. Pressure was generated using an infusion pressure cuff (D) (Sulz a.N., Germany) and connected with infusion tubes.

The second circuit consisted of Krebs buffer stained by methylene blue (MB) and was tempered to 37 °C. MB was added to the previously described Krebs buffer in a concentration of 1.4 mg/l corresponding to a common intravenous bolus injection of MB^25^. The applied pressure was increased by 10 mmHg every two minutes. As soon as the MB-stained Krebs buffer leaked, the peristaltic pump was switched off. At the end of the two minutes, the circuit (1) pump was reactivated to provide a fresh Krebs buffer to nourish the vessels and replace the MB Krebs buffer in the test chamber before the next two-minute pressure increase was performed. After each vessel was tested, the entire circuit (1) buffer was replaced.

Deficiencies in the anastomoses were graded modified to an established protocol^26^: (1) initial leakage from the anastomosis into the test chamber. (2) The leakage was defined as moderate if the colored Krebs buffer leaked at > 25% of the suture line or between three sutures or > 25% of the circular vessel surface covered with the VIVO. (3) Leakage was considered severe when the MB Krebs buffer leaked at > 50% of the suture line or between six sutures or > 50% of the circular vessel surface covered with VIVO. (4) Final anastomotic failure was defined as a vascular exit of 20 ml/min. At this rate, an anastomosis failure was assumed due to the high loss of stained Krebs buffer. The end of the test was determined at anastomosis failure or at an intraluminal pressure load of 300 mmHg. Pressure greater than 300 mmHg was far above physiological pressure and, therefore, not part of the test.

### Tensile strength determination

Twenty vessels from each group were tested for their longitudinal tensile strength using a standard tension testing machine (Zwick Z2.5, Zwick GmbH & Co, Ulm, Germany). The tests were conducted under normal climate conditions in compliance with DIN EN ISO 139. Air humidity was controlled at 65%, and a constant room temperature of 20 °C was maintained^21^. All samples were tested moistened with Krebs buffer solution and fixed at the top and bottom with secure mounting clamps. The specimens were clamped without any preload in accordance with the clinical setting (Fig. 5a). Axial force was applied continuously with a testing speed of two mm/min until the vessels ruptured.

### Statistical analysis

All data were evaluated using GraphPad Prism 7.0 (GraphPad Software, San Diego, USA). Parametric statistics were applied with data that met the D´Agostino & Pearson criteria and passed the Bartlett´s and Brown-Forsythe tests for equal variances. Relevant results were analyzed by one-way analysis of variance (ANOVA) for multiple comparisons followed by Tukey`s post hoc analysis. Time for anastomosis was analyzed when data hit the criteria of D´Agostino & Person normality test and passed F-test for variances. Relevant results were analyzed using an unpaired t-test. The Mann–Whitney-U-Test for nonparametric independent variables was used for burst pressure tests to compare the differences between parameters. All data represent the means ± SD. Statistical significance was determined when *p* ≤ 0.05.

## Results

**Figure 3:**
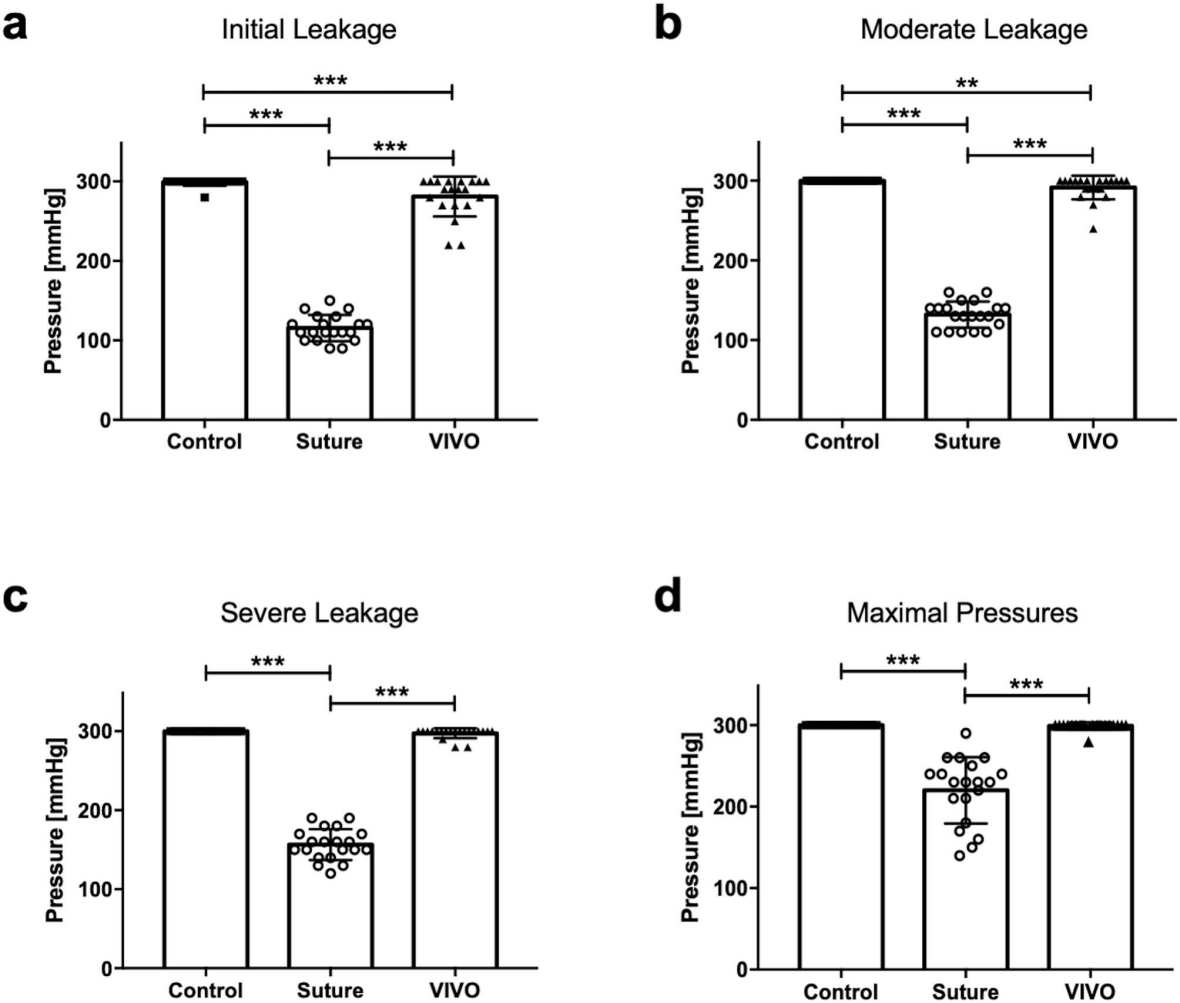
Scatter plot with bars of burst pressure test of all groups. The pressures at which initial leakage (**a**), moderate leakage (**b**), and severe leakage (**c**) were observed were significantly lower in the Suture group compared to the VIVO anastomoses. The maximum pressure values (**d**) were also significantly lower in the Suture group. The figures were created using GraphPad Prism 7.0. ***p ≤ 0.001; **p ≤ 0.003. All scatter plots represent the means ± SD.

**Figure 4:**
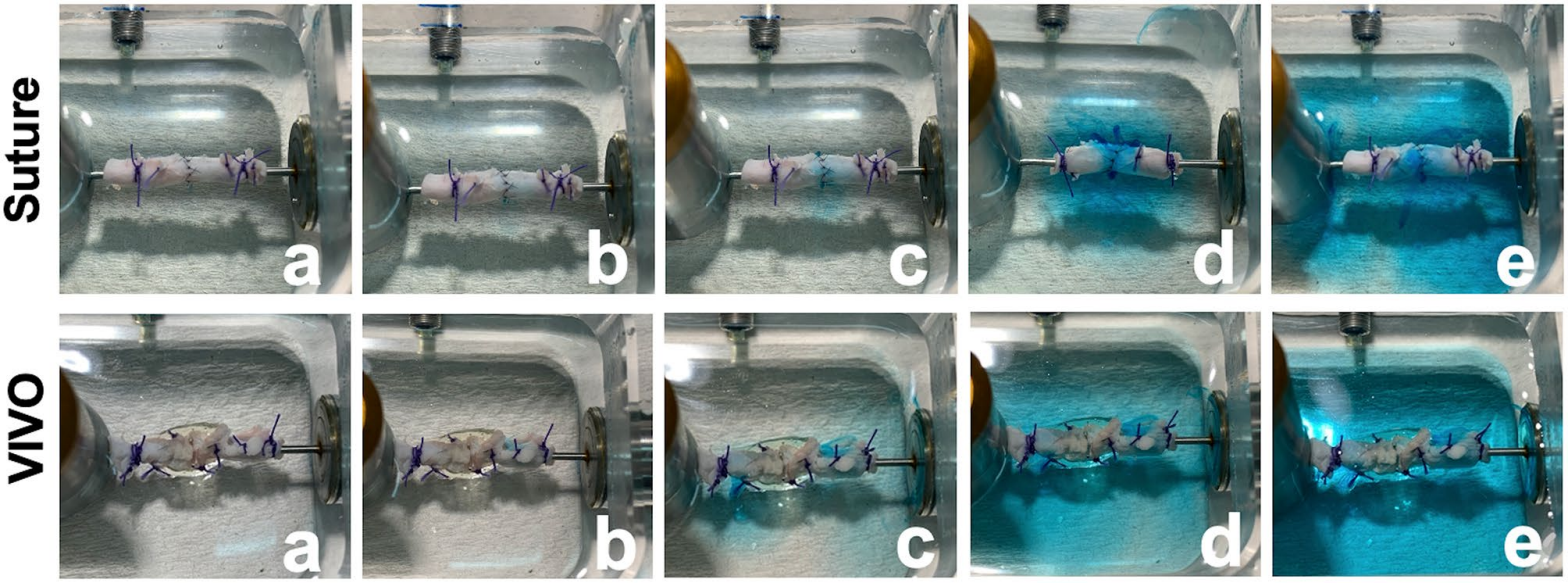
Image sequence of a representative burst pressure test of the Suture and VIVO group. The vessels are shown before pressure was applied (**a**). An initial leakage is visible in both anastomotic groups (**b**). Leakage > 25% of the anastomosis or leakage > 25% at of the circular vessel surface covered with VIVO (**c**). Leakage > 50% of or buffer leakage between 6 sutures or Krebs buffer leaked at > 50% of the circular vessel surface (**d**). The failure of anastomoses at 20 ml/min (**e**).

Table 2 shows the results of pressure burst tests for all tested groups. Apart from an initial leakage of one vessel at 280 [mmHg] from a side branch, no leakage was observed in the C group. Furthermore, VIVO anastomoses were significantly (p ≤ 0.001) superior to sutured anastomoses in terms of initial, moderate and severe leakage (Fig. 3). The failure of an anastomosis at 20 ml/min occurred within the VIVO group in two anastomoses at high pressures. Nevertheless, the mean value of the maximum pressure for this group was 299 ± 4.47 [mmHg] without differences to the control group but with significantly higher values (p ≤ 0.001) compared to sutured anastomoses. In contrast, anastomosis failure was observed in all sutured vessels and was determined at maximal pressures of 220 ± 40.65 [mmHg]. The time to create VIVO anastomoses 553.8 ± 82.44 [sec] was significantly faster (p ≤ 0.001) than sutured anastomoses of 1090 ± 32.6 [sec]. Representative sequences of images for burst pressure tests are shown in Fig. 4.

**Table 2:** Results of burst pressure test.

Groups	Initial leakage	Initial leakage (mmHg) Mean ± SD	Moderate leakage	Moderate leakage (mmHg) Mean ± SD	Severe leakage	Severe leakage (mmHg) Mean ± SD	Maximal pressure Mean ± SD	Failure anastomosis (20 ml/min)	Time for anastomosis (sec) Mean ± SD
C (n = 20)	1/20	299 ± 4.47	0/20	300 ± 0	0/20	300 ± 0	300 ± 0	0/20	N/A
Suture (n = 20)	20/20	115.5 ± 16.38	20/20	132 ± 16.42	20/20	156.5 ± 19.54	220 ± 40.65	20/20	1090 ± 32.6
VIVO (n = 20)	12/20	281 ± 25.11	8/20	291.5 ± 14.96	3/20	297.5 ± 6.39	299 ± 4.47	2/20	553.8 ± 82.44

**Figure 5:**
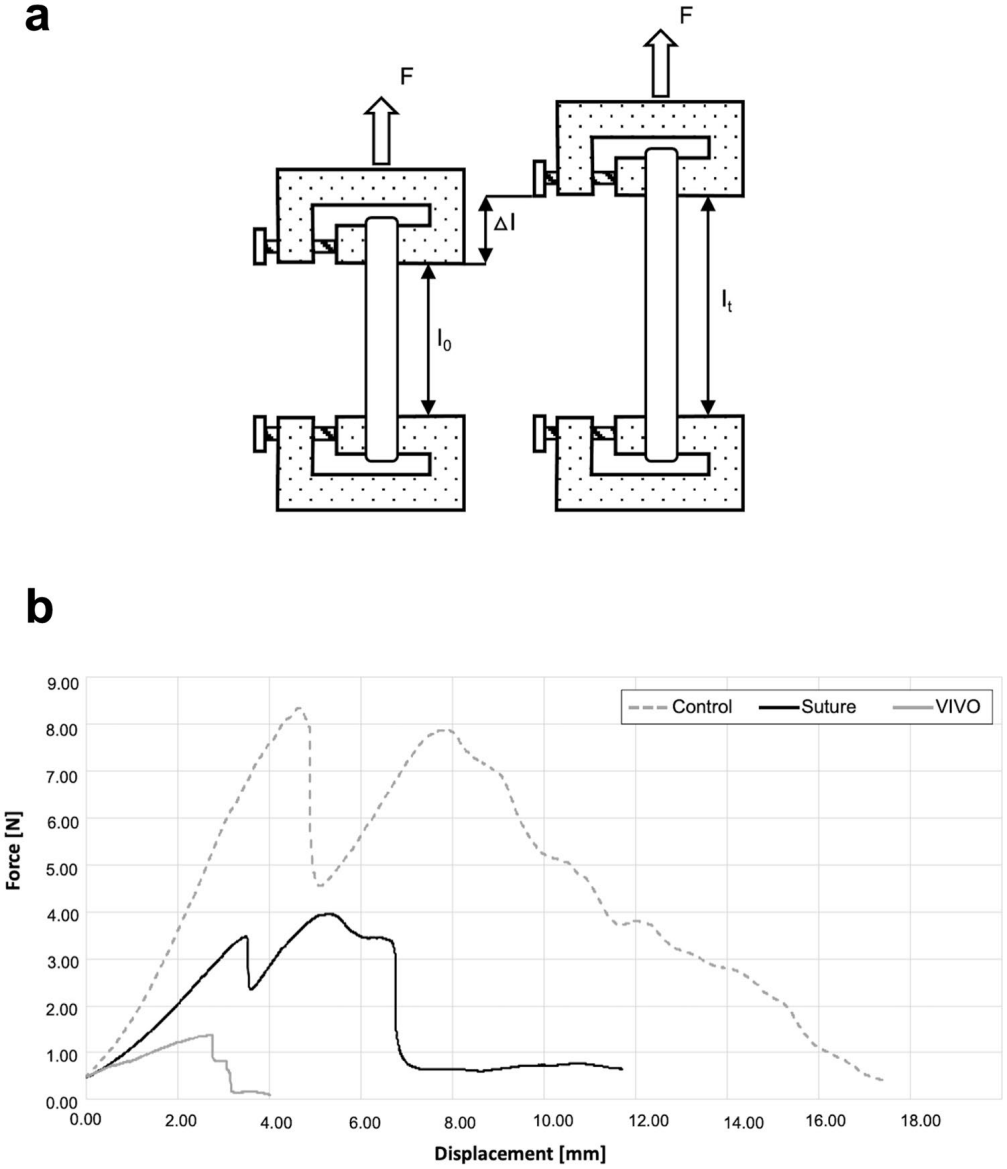
A Sketch of the standard gripping device for tensile strength determination in our test set-up with secure mounting clamps (**a**). The lower clamp is fixed, whereas the test force is applied to the upper clamp. The samples were clamped between these clamps. On the left is the device in its initial position with a length of I_0_. On the right is the specimen with an elongation of Δl in a defined time period l(t). Graphical illustrations of axial forces of characteristic specimens (**b**).

**Figure 6:**
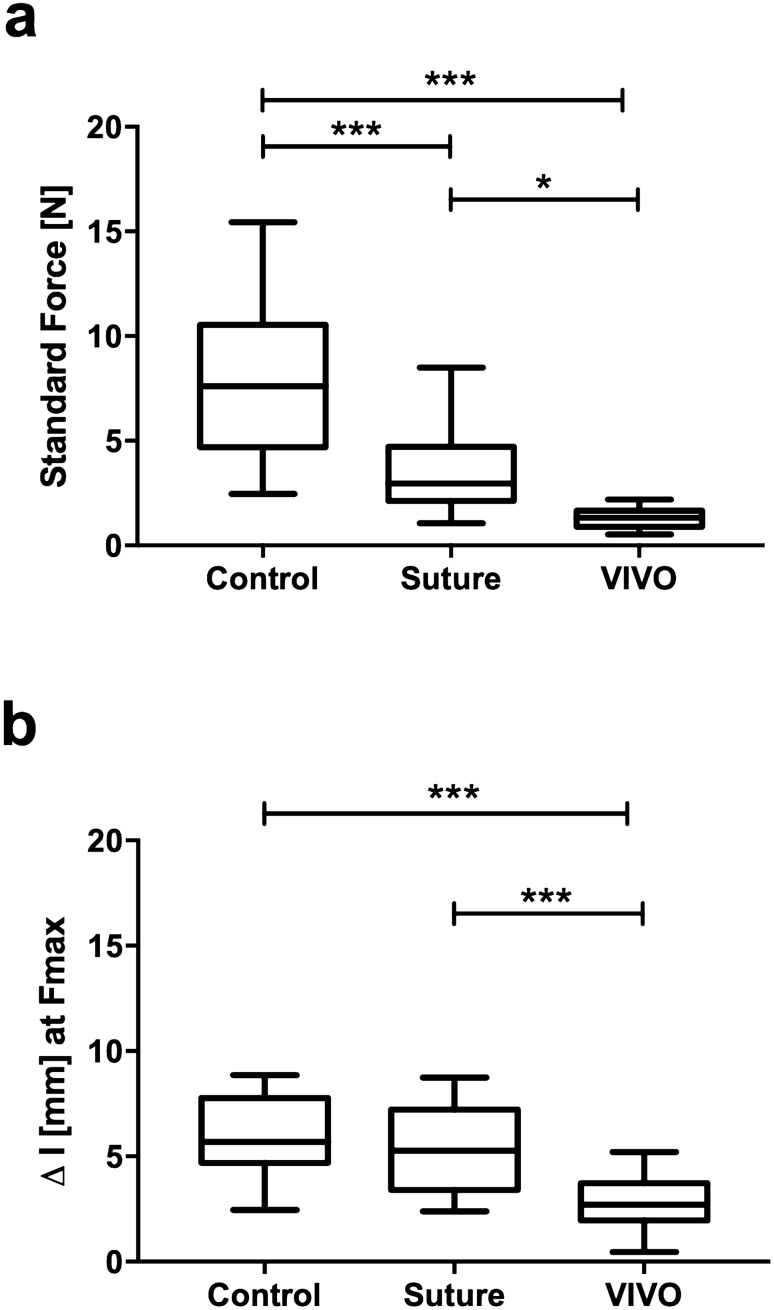
Box and whisker graphics of maximal tensile force and of Δl of all groups. The maximal tensile force of control vessels is the highest, followed by the Suture group (**a**). The comparison of Δl of all groups shows similar tendencies (**b**). ***p ≤ 0.001; *p ≤ 0.023. All scatter plots represent the means ± SD.

Table 3 shows the tensile test results of all three groups. As expected, group C served as a standard for artery tensile force and showed the tensile test’s highest values, with 7.85 ± 3.71 [N]. As our gold standard for anastomoses, the group of sutured vessels showed significantly higher (p ≤ 0.01) tensile forces at 3.64 ± 2.2 [N] compared to VIVO 1.33 ± 0.46 [N] (Figs. 5 and 6). The range of the first turning point of the Suture and VIVO curve correlated with the first dehiscence, which was observed visually. This dehiscence defined the clinically crucial moment of leakage and failure of the anastomosis.

**Table 3:** Results of tensile strength determination.

Groups	Tensile force Mean ± SD	Vascular rupture	Rupture of anastomosis	ΔI at Fmax Mean ± SD
C (n = 20)	7.85 ± 3.71	20/20	0/20	6.1 ± 1.86
Suture (n = 20)	3.64 ± 2.2	7/20	13/20	5.18 ± 2.06
VIVO (n = 20)	1.33 ± 0.46	0/20	20/20	2.75 ± 1.17

As expected, the vessel in group C, as the standard for arterial tensile force, showed the highest values. The applied forces rose steeply to a global maximum of 7.6 [N] at an elongation of 5.68 [mm]. A representative anastomosis of VIVO showed a constant increase up to a local maximum of 1.33 [N] at an extension of 2.7 [mm]. After this maximum, the force decrease was interrupted by a plateau.

## Discussion

Anastomoses with suture have become an integral part of modern reconstructive surgery and are a fixed component of many therapies for tissue deficits. Although they are time-consuming^27^ and success is user-dependent^28^, they are currently the method of choice. Therefore, the search for faster and easier methods has been an integral part of the science of microsurgery in recent years. We assume that the avoidance of manual suturing techniques, especially for inexperienced surgeons, will increase time efficiency and significantly reduce the rates of complication. Science is looking for reliable tissue adhesives that are fast, easy to use, have good tissue compatibility, and, above all, have sufficient adhesive strength in wet tissue^17,29,30^. Time is a particularly important factor in the duration of complex microsurgical procedures. A longer operation time in microsurgical procedures is associated with an increased risk of postoperative complications^31^. Significant reductions in operation time potentially reduce the risk of complications and provide the opportunity for cost savings^31–33^. Head et al. showed that microvascular devices are more expensive to purchase but result in shorter operation time due to ease of use, leading to a reduction in cost compared to hand-sutured anastomoses^33^. The adhesive-based anastomoses with an intervention time of 553.8 ± 82.44 [sec] unquestionably demonstrate the faster creation time of VIVO and easier surgical handling than sutured anastomoses. Although the novel adhesive is not yet available for purchase, it offers a potential and effective anastomotic procedure based on time savings, which needs to be carefully evaluated in a series of preclinical experiments.

Our results have shown that the use of VIVO provides a suture-free and, at the same time, a low-leakage form of anastomosis with a leakage rate of 0% at physiological pressures. In this group, initial leakage was observed at 281 ± 25.11 [mmHg]. The result was that, at unphysiologically high pressures, a tight bond between the vessel stumps was ensured.

The tissue adhesive VIVO showed good adhesion in mass bleeding and wet tissues^18,19^. In addition, our results show that VIVO has sufficient tensile strength 1.33 ± 0.46 [N] and burst pressure 299 ± 4.47 [mmHg], to adhere adequately to wet tissue, making it a suitable material for seamless vascular anastomoses.

Maintaining the blood supply is crucial and is particularly vulnerable during the first weeks after microsurgery. If a perfusing or draining vessel is occluded by thrombosis, wound healing, and integration of the graft in the recipient area can be compromised and lead to the loss of all or part of the transplant in up to 20% of cases^21,34^. Regardless of the type of tissue adhesive, vascular occlusions, and thromboembolic material were detected more frequently due to the use of adhesives during microvascular anastomosis^6^. Intraluminal devices such as stents, on the other hand, serve as intraluminal connection devices and can secure the opening of the vessels during the bonding process and prevent the penetration of adhesives into the vessel lumen.

A common approach is the use of intraluminal stents made of dissolving materials^35,36^ or metal stents^1,21^ to maintain vessel lumen patency. Although many experiments on the use of stents for microsurgical anastomoses have been reported, many limitations indicate poor usability and feasibility. The use of stents with a balloon catheter always requires a second incision site for anastomosis^1^. The design of the stent in this study allowed the stent to be squeezed and inserted into the vessel stumps using microsurgical forceps so that no additional access to the vessels was necessary.

Another limitation of stents is that they must be manufactured with desired geometries and dimensions, which do not allow their application when using anastomoses of unequal vascular lumina, which are common in reconstructive procedures. It is common for the vessel lumina to be interindividual and also differ between the graft vessel and the recipient vessel in reconstructive surgery^36^. As with many studies, one limitation of this research is the lack of a stent design and architecture that addresses the success and improvement in different vessel diameters.

Although VIVO showed the lowest tensile force of 1.33 ± 0.46 [N] compared to native vessels and sutured anastomoses this was still satisfactory in accordance to Colen et al.^37^ describing a mean force of 96.0 [N] in anastomoses with eight stitches. In comparison, the combination of VIVO and stent shows satisfactory results with a mean maximum value of 133.3 ± 45.57 [N] (1 N = 1 kg m/s^2^; acceleration due to gravity: 1 N = 1 kg 9.80665 m/s^2^)^21^.

Following completion, an anastomosis is exposed to increased longitudinal forces^38^. In end-to-end anastomoses, a rise in blood pressure leads to an exponential increase in vessel length. The strength of the longitudinal tensile forces acting on the anastomosis thus depends primarily on blood pressure^38^. In this case, VIVO application proved to be superior in terms of pressure for initial leakage, moderate leakage, and heavy leakage (p ≤ 0.001) compared to sutured anastomoses in the pressure test. These results reflect an effective anastomosis technique. However, with respect to the success of all anastomoses, un-physiological traction on the pedicles should be avoided intraoperatively and postoperatively. A tension-free position of the vessels in the situs guarantees the success of every anastomosis.

Preliminary test results show that the use of a pressure apparatus is valuable for testing the bursting pressure of native, sutured, and adhesive-based micro anastomoses. We demonstrated that the strong wet tissue surface adhesion, suitable mechanical properties, and ease of VIVO use are properties well suited to sutureless anastomoses.

A critical reflection on this study focused on the setting for the ex vivo experiment. The effects of forces caused by arterial vessel pulsations could not be determined under these strict test conditions. Thrombotic or hemodynamic effects could not be investigated due to the absence of blood. Furthermore, for a clinical application, knowledge about interactions with the coagulating components of blood and long-term investigations of degradation mechanisms, toxicity and compatibility of the immune system with VIVO are critical issues. Therefore, future studies addressing these parameters as well as the biocompatibility will be investigated in subsequent short-term, and long-term animal studies.

## Conclusions

Sutureless adhesive-based anastomoses have achieved very satisfactory results related to traction, have withstood unphysiological high pressures, and have proved superior to sutured anastomosis in addressing many technical issues. Therefore, future studies on these parameters as well as biocompatibility in further short-term and long-term animal studies will be needed to validate outcomes to confirm safety of the suture-free adhesive-based anastomosis.

## Data Availability

All data generated or analysed during this study are included in this published article.
